# Multiple microbiologic tests for tuberculosis improve diagnostic yield of bronchoscopy in medically complex patients

**DOI:** 10.12688/aasopenres.12980.1

**Published:** 2019-07-16

**Authors:** Dilshaad Fakey Khan, Moosa Suleman, Prinita Baijnath, Rubeshan Perumal, Vedanthi Moodley, Zoey Mhlane, Taryn Naidoo, Thumbi Ndung'u, Emily B. Wong

**Affiliations:** 1 Department of Pulmonology, Inkosi Albert Luthuli Central Hospital, Durban, KZN, 4000, South Africa; 2 Center for the AIDS Programme of Research in South Africa, Durban, South Africa; 3 Africa Health Research Institute, Durban, South Africa; 4 Division of Infection and Immunity, University College London, London, UK; 5 HIV Pathogenesis programme, Doris Duke Medical Research Institute, Durban, South Africa; 6 Max Planck Institute for Infection Biology, Berlin, Germany; 7 The Ragon Institute of Massachusetts General Hospital, Massachusetts Institute of Technology, Massachusetts, USA; 8 Division of Infectious Diseases, Massachusetts General Hospital, Boston, USA

**Keywords:** Bronchoalveolar lavage, Xpert MTB/RIF, GeneXpert, TB diagnostic

## Abstract

**Background: **Bronchoalveolar lavage (BAL) is indicated for medical evaluation of complex cases of lung disease.  There is limited data on the performance of tuberculosis (TB) microbiologic tests on BAL in such patients, particularly in human immunodeficiency virus (HIV) and TB endemic areas.

**Methods:** We evaluated the performance of *Mycobacterium tuberculosis* (Mtb) culture and up to two simultaneous Xpert MTB/RIF tests on BAL fluid against a consensus clinical diagnosis in 98 medically complex patients undergoing bronchoscopy over a two-year period in Durban, South Africa.

**Results: **TB was the most frequently diagnosed lung disease, found in 19 of 98 participants (19%) and was microbiologically proven in 14 of these (74%); 9 (47%) were culture positive and 5 were positive on at least one Xpert MTB/RIF assay. Immunosuppression prevalence was high (26% HIV-infected, 29% on immunosuppressive therapy and 4% on chemotherapy). Xpert MTB/RIF had low sensitivity (45%) and high specificity (99%) when assessed against the consensus clinical diagnosis. Compared to TB culture, a single Xpert MTB/RIF increased the diagnostic yield by 11% and a second Xpert MTB/RIF by a further 16%.

**Conclusion: **Although Xpert MTB/RIF had a low sensitivity, sending two tests improved the microbiologically-proven diagnostic yield of bronchoscopy from 47% to 74% compared to culture alone.

## Introduction

Globally, tuberculosis (TB) is the ninth leading cause of mortality with an estimated 1.6 million deaths reported in 2016 ^1^. South Africa has one of the world's highest burdens of TB with an estimated incidence of 438 000 cases in 2016 ^2^. The "End TB Strategy" aims to reduce global TB incidence by 90% and TB-related deaths by 80% by 2030 ^1^.

In 2011, the World Health Organization (WHO) recommended the GeneXpert MTB/RIF assay (Cepheid, Sunnyvale, CA, USA), a TB polymerase chain reaction (PCR) based test as the initial investigation of choice in patients with human immunodeficiency virus (HIV) co-infection or presumed multidrug resistant (MDR) TB ^3^. Despite the introduction of this widely available test with a rapid turnaround time into the South African public sector in 2011, TB remains notoriously difficult to diagnose, for reasons including suboptimal adherence to national diagnostic algorithms and challenges related to patient follow-up ^4, 5^.

Few data exist to guide the use of Xpert MTB/Rif on bronchoalveolar lavage (BAL) specimens. Xpert MTB/RIF has been evaluated in BAL specimens from individuals with presumed TB who are sputum scarce or smear negative and has been shown to outperform smear microscopy in both high and low TB burden regions ^6– 9^. The South African national TB guidelines do not specify whether BAL specimens should be processed similarly to sputum for the Xpert MTB/RIF test, how many specimens should be tested, whether the specimens should be centrifuged prior to processing, or how results should be interpreted in cases of non-concordance with other TB diagnostic tests ^10^.

In our public-sector based pulmonology clinic in urban South Africa, diagnostic bronchoscopy is used to evaluate medically complex patients for a variety of respiratory presentations occurring in the background of high rates of HIV and TB endemicity ^11^. In this context, mycobacterial smear and *Mycobacterium tuberculosis* (M.tb) culture are routinely performed on all diagnostic BAL specimens. In 2014, the use of Xpert MTB/RIF was approved for use on non-sputum samples at our facility. During this same period, a second Xpert MTB/RIF test was performed on most BAL samples obtained at our facility by the African Health Research Institute (AHRI) as part of an ongoing research study. Access to two Xpert MTB/RIF results performed on pooled BAL fluid sample at two independent laboratories provided a unique opportunity to evaluate the diagnostic yield of M.tb culture and multiple MTB/RIF assays against the clinical consensus diagnosis.

## Methods

### Study setting

Inkosi Albert Luthuli Central Hospital is a quaternary hospital in Durban, KwaZulu-Natal (KZN), South Africa. It is one of two hospitals in the public sector providing subspecialist services for the province. Patients with suspected infection (including TB), inflammatory lung disease or lung malignancy are referred to the Department of Pulmonology for flexible bronchoscopy and bronchoalveolar lavage (BAL) for diagnostic purposes. The diagnosis or exclusion of TB is an important aspect to the clinical management of these patients. Bacterial, mycobacterial and fungal smear and culture are performed routinely on all BAL specimens whilst cytology and tests for *Pneumocystis jerovicii* are performed only when clinically indicated. Since 2014, Xpert MTB/Rif has been performed routinely on all BAL specimens. Since 2013, patients undergoing diagnostic bronchoscopy at the facility have been offered enrollment in a research protocol, "Bronchoalveolar lavage fluid collection for the study of *Mycobacterium tuberculosis* immunology" (BE037/12).

### Study population

We retrospectively audited a database of all participants (≥18 years of age) who consented to participate in the above-mentioned study and had adequate return of bronchoalveolar lavage fluid for analysis between July 2014 and June 2016. Patients with at least one Xpert MTB/RIF result and a TB culture result were included.

### Bronchoscopy procedure

Standard bronchoscopy procedure was performed with appropriate sedation, monitoring of vital signs and clinician assessment regarding procedure safety ^12^. Accompanying chest radiography or computed tomography (CT) scans dictated the lung segment that was sampled. Two hundred millilitres of sterile saline was infused into the lung segment in 20 ml aliquots, with the lavage fluid pooled into a sterile container.

### Microbiological Tests

Bronchoalveolar fluid was sent for laboratory testing, including Xpert MTB/RIF [Catalogue number CGXMTB/RIF-50] (1 mL) and TB culture (5 mL in liquid culture medium for evaluation. The TB culture specimen was decontaminated with sodium hydroxide (1%), sodium citrate and PH 6.8 phosphate buffer, then added to the Mycobacteria Growth Indicator Tube (MGIT) [Catalogue number 245122], containing 4ml Middlebrook 7H9 broth liquid medium supplied by Becton Dickson. An antibiotic mixture (0.8ml) comprising Polymyxin B (6000 µg), Amphotericin B (600 µg), Nalidixic acid (2400 µg), Trimethoprim (600 µg) and Azlocillin (600 µg; PANTA; BBL MGIT PANTA Antibiotic mixture; BD)[Catalogue number 245124] was added and the specimen and innoculated into the BACTEC MGIT 960 system (Becton Dickson, Franklin Lakes, NJ, USA)[Catalogue number 445870]. A second Xpert MTB/RIF specimen (1 mL) was sent to the independent research laboratory. Both laboratories performed parallel testing of aliquots of the pooled samples using version 4.3 of the Xpert MTB/RIF assay according to the manufacturer specified protocol ^13^.

### Consensus diagnosis of pulmonary disease at the time of bronchoscopy

A panel of three clinicians (a general physician [DFK], an infectious disease specialist [EBW] and a pulmonologist [MS]) retrospectively analyzed each case utilizing all available clinical data (clinical history, physical examination, radiology, other laboratory and microbiology results, histopathology, results of all TB diagnostic tests, and data associated with follow-up visits). The panel determined the consensus diagnosis that best explained each participant's lung disease.

Participants were categorized as having a consensus diagnosis of tuberculosis if they had active pulmonary tuberculosis at the time of bronchoscopy, whether or not additional underlying lung pathology was present. Participants with a consensus diagnosis of tuberculosis were classified according to the updated WHO TB case definition as "bacteriologically confirmed" if there was any positive biological specimen (AFB smear microscopy, TB culture, or Xpert MTB/RIF) or "clinically diagnosed" if the TB microbiological tests were negative and the diagnosis was made based on other evidence (e.g., histology, response to anti-tuberculosis treatment) ^14^.

### Ethical considerations

All patients provided informed consent to participate in the research protocol, which was approved by the University of KwaZulu-Natal Biomedical Research Ethics Committee (BE610/16), the Partners Institutional Review Board and the KwaZulu-Natal Department of Health (KZ_2016RP53_969). Patients were allocated sequential numerical identity numbers, which are not medical identifiers.

### Statistics

Sensitivity and specificity for each test was calculated using the clinical consensus diagnosis as the gold standard, using cross-tabulation. All data was analysed using SPSS software (SPSS 25.0, Armonk NY: IBM Corp). Extracted data is available as underlying data ^15^.

## Results

### Cohort characteristics and consensus diagnosis

**Figure 1.**
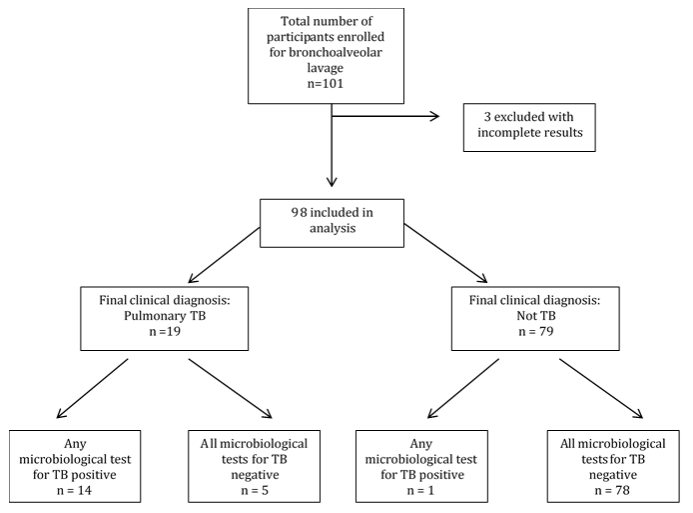
Study flow diagram showing the patients included in the analysis and proportion of patients with positive and negative bronchoalveolar lavage (BAL) tuberculosis microbiologic results.

**Figure 2.**
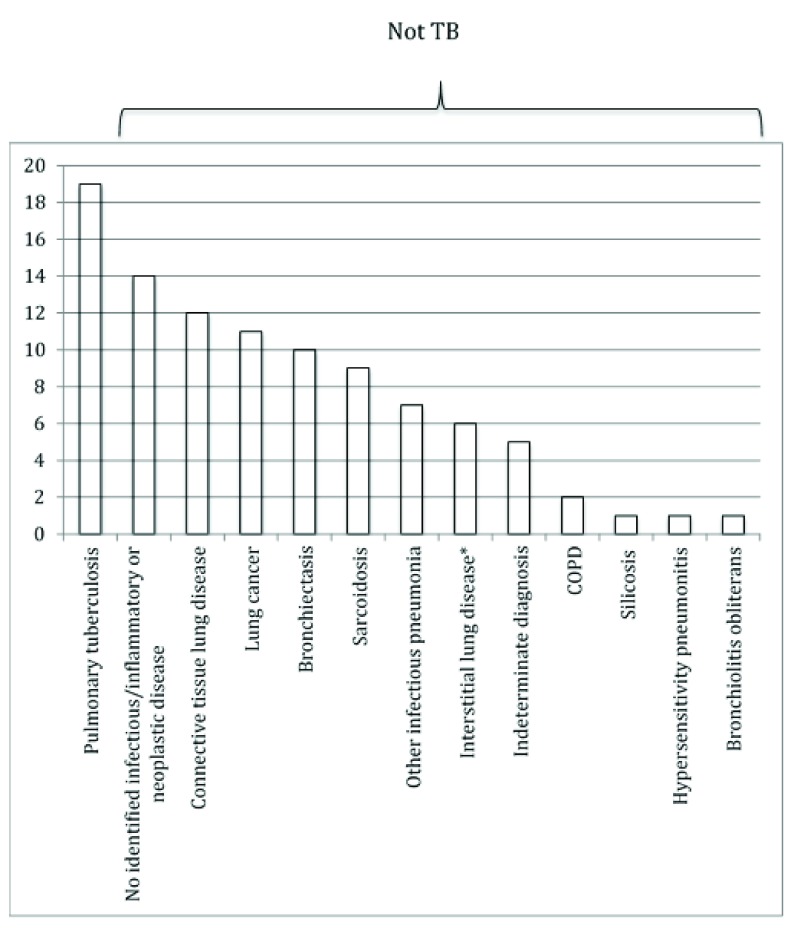
Clinical diagnosis (%) of the 98 patients enrolled in the study: Participants were categorized as having a consensus diagnosis of tuberculosis if they had active pulmonary tuberculosis at the time of bronchoscopy, whether or not additional underlying lung pathology was present. *Interstitial lung disease not due to connective tissue disease i.e Idiopathic pulmonary fibrosis, Non-specific interstitial pneumonia COPD – chronic obstructive pulmonary disease.

In total, 101 patients were enrolled in the parent study between July 2014 and June 2016; of these 98 had a BAL TB culture result and at least one BAL Xpert MTB/RIF result and were included in the final analysis ( Figure 1). The median age of the study participants was 48 years (interquartile range 19-80 years) and 51% of the subjects were female. Of these, 19 participants had a consensus diagnosis of tuberculosis at the time of bronchoscopy (19%). A consensus diagnosis that did not include active tuberculosis was indicated for 79 participants (81%); of these, the leading diagnoses were: no identified infectious/inflammatory/neoplastic lung disease, interstitial lung disease associated with connective tissue disease, lung cancer, sarcoidosis and bronchiectasis ( Figure 2).

Immunosuppression prevalence was high. Of the overall cohort, 26% (25 participants) were HIV infected with 84% of those on antiretroviral therapy. Additionally, 29% were actively receiving immunosuppressive medication for connective tissue disease or sarcoidosis. Four percent were actively using or had recently received chemotherapy for solid organ or haematological malignancy.

Rates of previous episodes of tuberculosis were high overall (33%) and equally balanced between participants with current TB and in participants who did not have a current episode of active TB (32% and 33% of each group respectively) by clinical consensus diagnosis.

### Performance of TB diagnostic tests against the consensus clinical diagnosis

Of the 19 participants with a consensus diagnosis of tuberculosis, 14 (74%) had bacteriologically proven TB ( Table 1). M.tb culture provided the basis for bacteriologic diagnosis of TB in nine (47%) of these cases, two thirds (7/9) of which were also supported by at least one positive Xpert MTB/RIF test. In the 10 cases with negative M.tb culture, at least one positive Xpert MTB/RIF test provided the basis for bacteriologic diagnosis in five of the cases. Five cases (26%) had a clinical consensus diagnosis of TB with all bacteriologic tests negative. Compared to the diagnostic yield of M.tb culture alone (47%), the addition of one Xpert MTB/RIF test increased the yield by two cases (11%), with a second Xpert/RIF adding three additional cases (16%).

**Table 1.** Analysis of Pulmonary tuberculosis cases classified according to the WHO TB case definitions: Patients grouped (A–C) based on strength and concordance of TB microbiologic tests.

TB diagnostic tests in 19 patients with Tuberculosis *(All tests performed on bronchoalveolar lavage unless otherwise specified)*				
PID number	TB Culture	Xpert MTB/Rif 1	Xpert MTB/Rif 2	Other diagnostic test
*Group A: TB culture positive*				
1020	+	+	ND	
1025	+	+	ND	
1036	+	+	ND	
1060	+	+	+	
1084	+	+	+	
1026	+	+	-	
1030	+	-	ND	
1059	+	-	-	
1057	- *	-	-	
*Group B: TB culture negative with at least one positive Xpert MTB/Rif*				
1007	-	+	ND	
1016	-	+	ND	
1047	-	-	+	
1072	-	-	+	
1093	-	-	+	
*Group C: TB culture and Xpert MTB/Rif negative*				
1032	-	-	-	Histology: AFB + on open lung biopsy
1055	-	-	-	Miliary pattern on CXR
1002	-	-	ND	
1005	-	-	ND	
1028	-	-	ND	

ND: Not done, AFB: Acid fast bacilli, CXR: Chest X-Ray. * Sputum culture positive one week prior to bronchoscopy.

Against the clinical consensus diagnosis, the sensitivity and specificity of M.tb culture alone was 47% and 100%, the pooled sensitivity and specificity of Xpert MTB/RIF was 45% and 99% and the sensitivity and specificity of all bacteriological tests compared to the clinical consensus diagnosis was 68% and 97% respectively.

Of the 19 cases with a clinical consensus diagnosis of TB, 12 (63%) had at least one negative BAL Xpert MTB/RIF resulting in a high false negative rate of 55%. Low bacillary load appeared to be a factor as 11 of these 12 cases (92%) were acid fast bacilli (AFB) negative on smear microscopy of the alveolar fluid.

There was one false positive BAL Xpert MTB/RIF result (1%) in our cohort. This case was determined to be due to laboratory error as the leftover sample was retested (due to low clinical suspicion of TB) and produced consistently negative results.

### Discrepancy between parallel Xpert MTB/Rif tests

Of the 98 patients enrolled, 63 participants (64%) had two BAL Xpert MTB/RIF results. Of those, 58 (92%) were concordant and five (8%) were discordant. Four of the discordant test pairs occurred in patients with a clinical consensus diagnosis of TB.

## Discussion

In this medically complex cohort of patients with high rates of HIV-infection and comorbidities necessitating therapeutic immunosuppression, TB was the single leading cause of lung disease at the time of bronchoscopy.

Despite intensive investigations including, in most cases, multiple Xpert MTB/RIF tests on bronchoalveolar lavage fluid, 26% of cases determined to have active tuberculosis by clinical consensus had no microbiological evidence for TB and less than half of the TB cases (47%) had a positive BAL TB culture. Performing Xpert MTB/RIF testing on bronchoalveolar lavage fluid increased the yield of bacteriologically proven TB cases and the yield was further increased by performing two independent tests.

In our cohort, the pooled sensitivity of Xpert MTB/RIF testing of BAL fluid compared to consensus diagnosis (45%) was lower than in other published reports from high-burden TB settings ^6, 7, 9, 16^. This is likely due to differences in the nature of our cohort as TB was not the primary diagnostic consideration for most of the participants we evaluated. Our findings are comparable to those of le Palud *et al* who evaluated the accuracy of Xpert MTB/RIF against a composite reference standard in 162 patients undergoing flexible bronchoscopy for presumed TB in a low TB burden country ^7^. They found Xpert MTB/RIF sensitivity to be 60% against the composite reference standard ^7^.

Most of the participants in our cohort who had a clinical diagnosis of TB had negative BAL AFB smears, low rates of M.tb culture positivity and high rates of conditions associated with paucibacillary disease including HIV-infection, connective tissue disease and/or therapeutic immunosuppression. Patients with extrapulmonary TB or HIV and TB co-infection who have culture negative TB are reported to have a lower bacillary load in the lungs attributed to poor cavity formation ^17– 19^ and lower likelihood of Xpert MTB/RIF positivity ^16^. We suspect that these factors contributed to the poor sensitivity of TB microbiological tests and specifically Xpert MTB/RIF in our cohort. In addition to factors associated with paucibacillary disease, differences in lung sampling and sample dilution can lower TB bacillary load in BAL fluid compared to expectorated sputum ^16^.

We found that multiple Xpert MTB/RIF testing on bronchoalveolar lavage fluid increased the yield of bacteriologically proven TB, consistent with the results of Boehme and colleagues, who conducted a multicenter study and found that testing multiple sputum samples with Xpert MTB/RIF had a modest benefit over a single test. In smear negative but culture confirmed TB, the sensitivity of Xpert MTB/RIF was 72% for one test, 85% for two tests and 90.2% for three tests ^20^. In contrast, Theron *et al* evaluated 154 patients with presumed TB, with two Xpert MTB/RIF assays on BAL fluid specimens (centrifuged and uncentrifuged) and found that centrifugation of a second Xpert MTB/RIF did not alter test sensitivity against TB culture ^9^.

In our cohort, the addition of a second Xpert MTB/RIF test resulted in a modest increase in non-concordance (8% of the 63 cases with two Xpert MTB/RIF tests) and of these, a significant proportion (80%) were deemed to be a true positive paired with a false negative result. We had a low BAL Xpert MTB/RIF false positive rate (1%). This did not significantly impact the test specificity and is comparable with results from other studies ^7, 9, 16^.

### Limitations

Patients evaluated at our hospital were medically complex and reflect a referral bias, making comparisons with other studies on Xpert MTB/RIF use in BAL fluid difficult. The use of two laboratories may have contributed to the discordance through procedural variability, despite the use of manufacturer specified methods. Finally, the numbers of participants with specific patterns of non-concordant TB test results were insufficient for specific analyses of causes contributing to the discordance.

## Conclusions

Our study shows that in a medically complex group of patients from a high HIV and TB endemic setting, TB remains the leading cause of lung disease but a significant proportion of patients have paucibacillary disease lowering the sensitivity of M.tb diagnostic tests. In this setting, negative TB diagnostic tests should be interpreted with caution. We urge clinicians to consider submitting additional Xpert MTB/RIF tests in patients with a clinical suspicion of TB as we have shown an improved yield of 11% with a single Xpert MTB/RIF and a further 16% with a second Xpert MTB/RIF assay. We recommend that when conducting a diagnostic evaluation of such patients, BAL testing should be augmented by sending additional specimens that may further increase diagnostic yield. Submission of expectorated or induced sputum or pooled endotracheal aspirate alongside BAL fluid has the potential to improve diagnostic yield ( Box 1). Additional research to improve the sensitivity of TB diagnostics in medically complex patients is urgently needed.

We acknowledge that for most clinicians working in the public sector in resource-constrained environments, access to CT scans and specialized procedures such as bronchoscopy or surgical lung biopsies may be unavailable, but we emphasize the importance of maintaining a high suspicion for tuberculosis and implementing good clinical judgment, even in the face of negative Xpert MTB/RIF tests. Many patients will need to be treated empirically for TB, but should receive vigilant follow-up to monitor for response to treatment.

Box 1. **Summary of the factors to consider when ordering and interpreting TB diagnostic tests**

Factors to consider:

- Patient characteristics: HIV status, immunosuppression (including the use of immunosuppressive drugs), previous tuberculosis (TB) and exposure to anti-tuberculous therapy
- Disease characteristics: Paucibacillary disease
- Sampling factors: Expectorated sputum vs bronchoalveolar lavage fluid

Recommendations:

- Attempt to obtain expectorated sputum samples or pooled endotracheal aspirates at bronchoscopy
- Submit two Xpert MTB/Rif tests
- When results are discordant, a positive result should be favoured and treatment for TB commenced
- Interpret negative TB diagnostic tests with caution in a high burden TB setting.

## Data availability

### Underlying data

Figshare: FakeyKhan_dataset.xlsx. https://doi.org/10.6084/m9.figshare.8174597.v1 ^15^

This project contains the following underlying data:

- FakeyKhan_dataset.xlsx (Participant age, HIV status, Previous TB history, current TB details, medical co-morbidities, Results of Xpert MTB/RIF test on BAL from 1. Research unit and 2. IALCH, BAL TB culture result, Other diagnostic test results of importance, final consensus clinical diagnosis)
- FakeyKhan_datadictionary.xlsx (Format and description of variables included in FakeyKhan_dataset.xlsx)

Data are available under the terms of the Creative Commons Attribution 4.0 International license (CC-BY 4.0).
