# Supplementary material for: Multiple microbiologic tests for tuberculosis improve diagnostic yield of bronchoscopy in medically complex patients
Source: AAS Open Res. 2019 Jul 16;2:25. [Version 1] doi: 10.12688/aasopenres.12980.1 (PMC7194149; doi:10.12688/aasopenres.12980.1)
Supplement: Peer review history

# Peer review: Reviewer response for version 1

**DOI:** 10.21956/aasopenres.14061.r27225

**Published:** 2019-10-30

**Authors:** Katalin A. Wilkinson

**Affiliations:** 1 Wellcome Centre for Infectious Diseases Research in Africa, Institute of Infectious Disease and Molecular Medicine, University of Cape Town, Cape Town, South Africa; 2 The Francis Crick Institute, London, UK

This report describes the use of culture and GeneXpert for *M. tuberculosis *in 101 patients undergoing diagnostic bronchoscopy. Of the 98 patients with BAL TB culture results and at least one BAL Xpert MTB/RIF result, 19 were found to have pulmonary TB.

 Comments:

1. Two independent laboratories performed parallel Xpert MTB/RIF testing of different aliquots (1 ml) of the pooled BAL sample. This is not biological replicate testing but rather a technical replicate analysis and therefore questions arise when the two labs find different results. The authors should comment on this, since it affects the conclusions.
2. As mentioned in the Results section, immunosuppression prevalence was high, including 25 participants (26%) with HIV infection. This group should undergo a subanalysis, especially regarding clinical diagnosis. ART duration would also be of interest. Additionally, the 19 pulmonary TB patients should also be analysed according to HIV status.

I confirm that I have read this submission and believe that I have an appropriate level of expertise to confirm that it is of an acceptable scientific standard.

# Peer review: Reviewer response for version 1

**DOI:** 10.21956/aasopenres.14061.r27057

**Published:** 2019-08-12

**Authors:** Henry C. Mwandumba

**Affiliations:** 1 Malawi-Liverpool-Wellcome Trust Clinical Research Program, University of Malawi College of Medicine, Blantyre, Malawi

The report by Khan *et al.* describes the use of multiple microbiologic tests for tuberculosis to improve the diagnostic yield of bronchoscopy in patients with clinical and radiological complex lung diseases. Overall, the report is written well in grammatically sound English.

Strengths:

1. Use of multiple diagnostic tests (smear microscopy, culture and GeneXpert).
2. Use of deep airway samples.
3. Comparison of the performance of the GeneXpert platform between 2 laboratories.

Weaknesses:

1. Retrospective study although original data collection appears to have been fairly robust.
2. It is not clear what the clinical implication of the study findings are. What number and type of tests do the authors recommend in "multiple microbiologic tests"? In the current study, GeneXpert is the only test that was performed twice by two different labs. In settings where resources are available, sputum/BAL smear microscopy, culture and GeneXpert would normally be performed for TB diagnosis, especially in patients with complex lung diseases.
3. It appears post-bronchoscopy sputum samples were not collected for TB diagnosis. This is an important sample and acts like induced sputum for TB diagnosis, usually better than BAL.
4. The term "clinical consensus diagnosis as the gold standard" only applied to this study as it is based on the opinions of the clinicians involved so the authors should make that clear. In TB diagnostics, culture is the gold standard.
5. Please revise the spelling of Becton and Dickinson under Methods.

I confirm that I have read this submission and believe that I have an appropriate level of expertise to confirm that it is of an acceptable scientific standard.
